# Comparison of the induced fields using different coil configurations during deep transcranial magnetic stimulation

**DOI:** 10.1371/journal.pone.0178422

**Published:** 2017-06-06

**Authors:** Mai Lu, Shoogo Ueno

**Affiliations:** 1 Key Lab. of Opt-Electronic Technology and Intelligent Control of Ministry of Education, Lanzhou Jiaotong University, Lanzhou, 730070, Gansu Province, P. R. China; 2 Department of Applied Quantum Physics, Graduate School of Engineering, Kyushu University, Fukuoka 812-8581, Japan; Nanjing Normal University, CHINA

## Abstract

Stimulation of deeper brain structures by transcranial magnetic stimulation (TMS) plays a role in the study of reward and motivation mechanisms, which may be beneficial in the treatment of several neurological and psychiatric disorders. However, electric field distributions induced in the brain by deep transcranial magnetic stimulation (dTMS) are still unknown. In this paper, the double cone coil, H-coil and Halo-circular assembly (HCA) coil which have been proposed for dTMS have been numerically designed. The distributions of magnetic flux density, induced electric field in an anatomically based realistic head model by applying the dTMS coils were numerically calculated by the impedance method. Results were compared with that of standard figure-of-eight (Fo8) coil. Simulation results show that double cone, H- and HCA coils have significantly deep field penetration compared to the conventional Fo8 coil, at the expense of induced higher and wider spread electrical fields in superficial cortical regions. Double cone and HCA coils have better ability to stimulate deep brain subregions compared to that of the H-coil. In the mean time, both double cone and HCA coils increase risk for optical nerve excitation. Our results suggest although the dTMS coils offer new tool with potential for both research and clinical applications for psychiatric and neurological disorders associated with dysfunctions of deep brain regions, the selection of the most suitable coil settings for a specific clinical application should be based on a balanced evaluation between stimulation depth and focality.

## Introduction

Transcranial magnetic stimulation (TMS) is a technique to stimulate the brain noninvasively. Magnetic fields are produced by passing a strong current through an electromagnetic coil placed upon the scalp that in turn induce electric field and eddy-currents in the underlying cortical tissue, thereby producing a localized axonal depolarization. TMS has become a major tool in brain research and the treatment of various psychiatric and neurological disorders [1–3]. In principle, any brain-related disorder that is associated with pathological activity of specific brain sites may be treated by this method. Treatment of depression was the first major therapeutic goal set for TMS [4]. Other potential applications include schizophrenia [5], Parkinson’s disease [6], Alzheimer’s disease [7], and various addictions [8–11]. In recent years, it has been demonstrated that depression is a disease affecting multiple brain regions [12, 13], which are associated with reward circuits [14, 15], such as the nucleus accumbens (NA), the ventral tegmentum area (VTA), amygdala, and medial prefrontal, cingulate. These brain areas normally lie at depths of approximately 6–7 cm. The standard TMS with round [16] and Fo8 [17] coils has been shown to be effective in the treatment of depression with a common target in the dorsolateral prefrontal cortex (DLPFC), which was assumed to be at the depth 2–2.5 cm from the surface of the head. The size of the magnetic field generated by this technique is not sufficient to reach the deeper cortical, subcortical and limbic areas. For this reason, despite the fact that superficial TMS has proved to be moderately effective in treating drug-resistant depression, it is hypothesized that deep TMS stimuli, which reach a much greater depth, may be more effective in increasing the antidepressant effect. To stimulate deeper neuronal regions such as reward-related pathways directly using traditional TMS, much higher stimulation intensities are needed, as the electric field decreases rapidly as a function of tissue depth. However, even if stimulation intensities could be highly increased at the source, the use of standard TMS at such high stimulation intensities does not allow safe stimulation and can lead to undesirable side effects. These limitations have led to the development of novel coil designs suitable for dTMS, which allows direct stimulation of much larger and deeper brain regions by significant reduction of the decay rate. In the past decade, there are several coil configurations potentially suitable for dTMS: double cone, H- and HCA coils.

The double cone coil can be considered as a larger Fo8 coil with an fixed angle of about 95 degree between the two wings [18]. Stimulation of regions at depth of 3–4 cm, such as the leg motor area, can be achieved using the double cone coil [19]. It has also been used for direct activation of the pelvic floor and lower limb motor representation at the interhemispheric fissure, as well as for transsynaptic activation of the anterior cingulate cortex via stimulation of the medial frontal cortex [20].

Another coil design for dTMS is called H-coil [21–24]. The H-coil was composed of a base portion running tangential to the scalp and return portions removed from the head. The coil with complicated winding patterns and larger dimensions is designed to generate summation of the electric field in a specific brain region at a depth of 4–6 cm by locating coil elements at different locations around this region, all of which have a common current component which induce electric field in the desired direction.

A family of dTMS coil designs called Halo coil, a large circular coil capable of being placed around the head was developed to provide subthreshold electric field in deep brain tissues. It has been proposed to work with the conventional circular coil at the top of the head. The Halo-circular assembly coil (HCA coil) enables the stimulation of the brain at greater depth with greater flexibility than is currently achievable with the conventional round coil [25, 26]. In order to stimulate deeper brain regions while decreasing the electrical field in superficial cortical regions, the coaxial circular (CC) coils was also developed [27, 28].

Among the dTMS coil design mentioned above, both double cone and H-coils have been applied in the practical clinical applications [29–31]. However, no study has been implemented to compare the field distributions in deep brain tissues by double cone, H-, and HCA coils in the same realistic head model. In the present work, we used the impedance method to numerically calculate the electric field induced in a realistic head model by employing the double cone, H- and HCA coils, and results were compared with that of conventional Fo8 coil. We first examined the characteristics of magnetic flux density (B-field) in head model by employing all four coils and investigated the B-field characteristics in deep brain subregions. Then we analysed the impact of coil configurations on the induced electric field (E-field) in the surface of gray matter (GM) and white matter (WM), as well as in deep brain subregions. Since the coils, especially the H- and HCA coils were placed around the head, close to both eyebrows, safety concern about the excitation of visual tissues was therefore arisen. We therefore focused on investigating the induced E-field in eyeball tissues and optical nerve by using different coil designs.

## Methods

### Coil design

**Fig 1:**
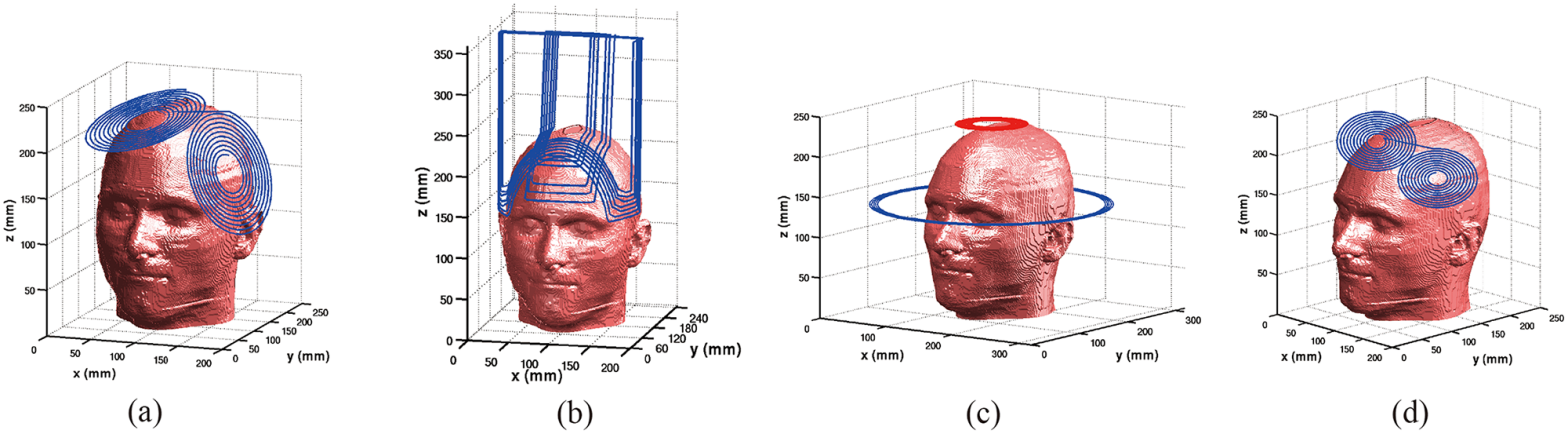
Realistic head model with coils (a) Double cone coil, (b) H-coil, (c) HCA coil and (d) Fo8 coil.

Three coil designs for dTMS have been numerically designed as shown in Fig 1, where Fig 1(a)–1(c) show the double cone coil, H-coil and Halo coil which are placed on the surface of the head model. For comparison, Fig 1(d) shows the modelled Fo8 coil.

The double cone coil was composed with two large circular coils with a fixed angle (95 deg) between them. The inner and outer radii of the circular wings are 20 mm and 70 mm, respectively. The number of the wire turns in each wing is 10. The H-coil was composed of base and return portions. The coil is designed to minimize the unintended stimulation of portions of the brain, while reducing the accumulation of the surface charges. The Halo coil with 5 turns has inner and outer radii of 138 and 150mm, respectively. It is operated simultaneously with a typical circular coil of mean diameter 90 mm and 14 turns located 100 mm above the Halo coil. For comparison, we have also modeled the figure-of-eight coil. The inner and outer radii of the circular wings are 10 mm and 50 mm, respectively. The number of the wire turns in each wing is 10. The same pulse currents with amplitude of I = 5.0 kA and working frequency 2.381 kHz was fed into each of the four coils.

### Realistic head model

**Fig 2:**
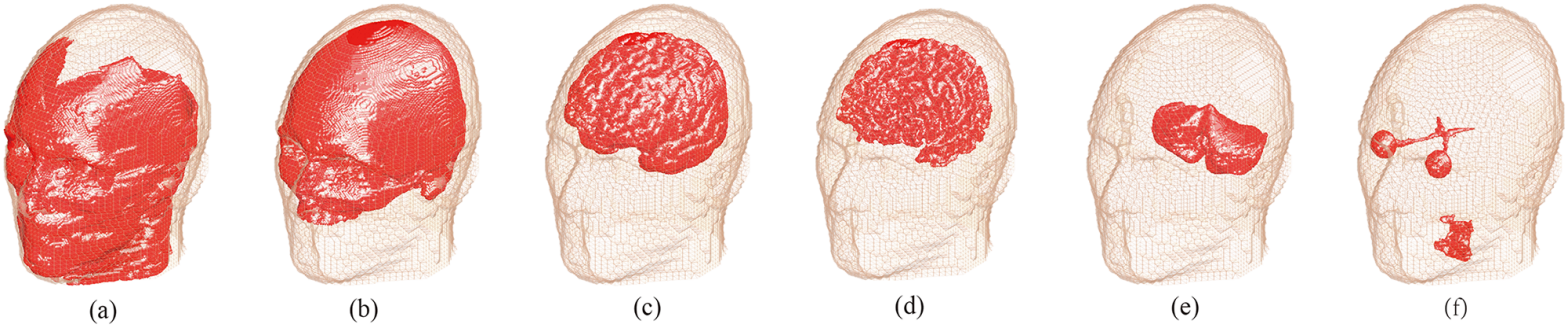
Transparency of the head tissues (a) Muscle, (b) Skull, (c) Gray matter (d)White matter, (e) Cerebellum and (f) Eye ball with visual nerves.

The realistic head model was obtained from a man model (Duke, 34-year-old male) developed by Virtual Family project [32]. The head model consists of 36 different kind of tissues, including several important deep brain regions, such as thalamus, hippocampus, pons, etc. The head model is composed of 10 million cubic voxels with resolution of 1mm. Fig 2 shows the head model with transparency of muscle, skull, GM, WM, cerebellum and visual tissues.

### Tissue conductivity and numerical method

The electrical conductivity of head tissues are modeled using the four-Cole-Cole model [33]. In this model, the head tissues subject to an electric field with angular frequency is modelled by relaxation theory and tissue conductivity can be calculated by fitting to experimental measurements [34–36]. In the present head model, since the number of tissue type is more than that in original Gabriel list [36], various tissues in the head model have been simulated with conductivities of similar tissues (i.e. thalamus, hippocampus, pons, etc have the same conductivity as that of brain grey matter). The conductivities of head tissues used in the simulations are shown in Table 1.

**Table 1:** Tissue conductivities (*f* = 2381 Hz).

Tissue	Conductivity *σ*[*S*/*m*]	Tissue	Conductivity *σ*[*S*/*m*]
Artery	7.00e-01	Hypothalamus	5.26e-01
Blood. Vessel	3.10e-01	Mandible	2.03e-02
Cartilage	1.75e-01	Marrow-bone	2.44e-03
Cerebellum	1.24e-01	MO*^3^	4.65e-01
CSF	2.00e+00	Midbrain	4.65e-01
CA*^1^	6.44e-02	Mucosa	8.46e-04
CP*^2^	6.44e-02	Muscle	3.31e-01
Connective-tissue	2.04e-01	Nerve	3.04e-02
Ear-cartilage	1.75e-01	Pineal-body	5.26e-01
Ear-skin	2.00e-04	Pons	4.65e-01
Eye-cornea	4.25e-01	Skin	2.00e-04
Eye-lens	3.31e-01	Skull	2.03e-02
Eye-sclera	5.07e-01	Spinal-cord	3.04e-02
Eye-vitreous-humor	1.50e+00	Teeth	2.03e-02
FAT	2.32e-02	Thalamus	1.04e-01
Gray matter	1.04e-01	Tongue	2.76e-01
Hippocampus	1.04e-01	Vein	7.00e-01
Hypophysis	5.26e-01	White Matter	6.44e-02

*^1^CA: Commissura-anterior;

*^2^CP: Commissura-posterior;

*^3^MO: Medulla-oblongata.

The head model was described using a uniform 3D Cartesian grid and is composed of 10 million cubic voxels. Assuming that the electric conductivities are isotropic and constant in all direction in each voxel, the head model is represented as a 3D network of impedances. The magnetic fields and the induced electric fields were calculated using the impedance method [37–39]. we have successfully employed the impedance method in the modelling of brain stimulation and electromagnetic dosimetry [40, 41].

## Results

**Fig 3:**
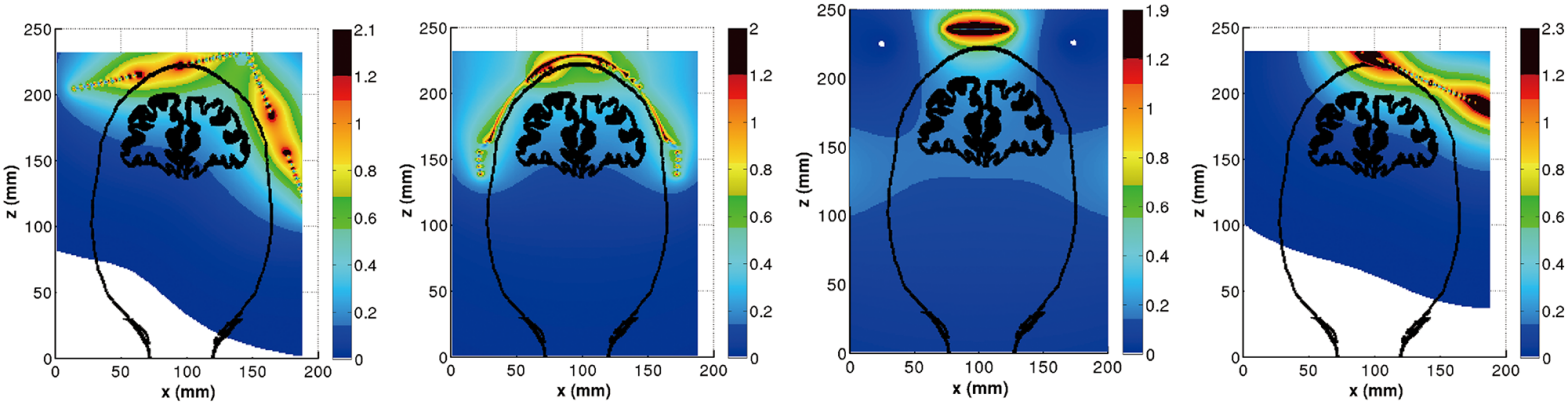
Distribution of B-field (Tesla) with the contour outline of scalp and GM in the coronal slice of y = 80 mm. (a) Double cone coil, (b) H-coil, (c) HCA coil and (d) Fo8 coil.

The distribution of B-field in the coronal slice (y = 80 mm) for double cone, H-, HCA and Fo8 coils are shown in Fig 3(a)–3(d). The contour outlines of scalp and gray matter were also included in each of the figures. As expected, higher magnetic field is presented on the coil surface. However, the B-field decays quickly. It was found the stimulation depth in the brain were significantly increased by double cone, H- and HCA coil (Fig 3(a)–3(c)) compared to that by Fo8 coil (Fig 3(d)).

**Fig 4:**
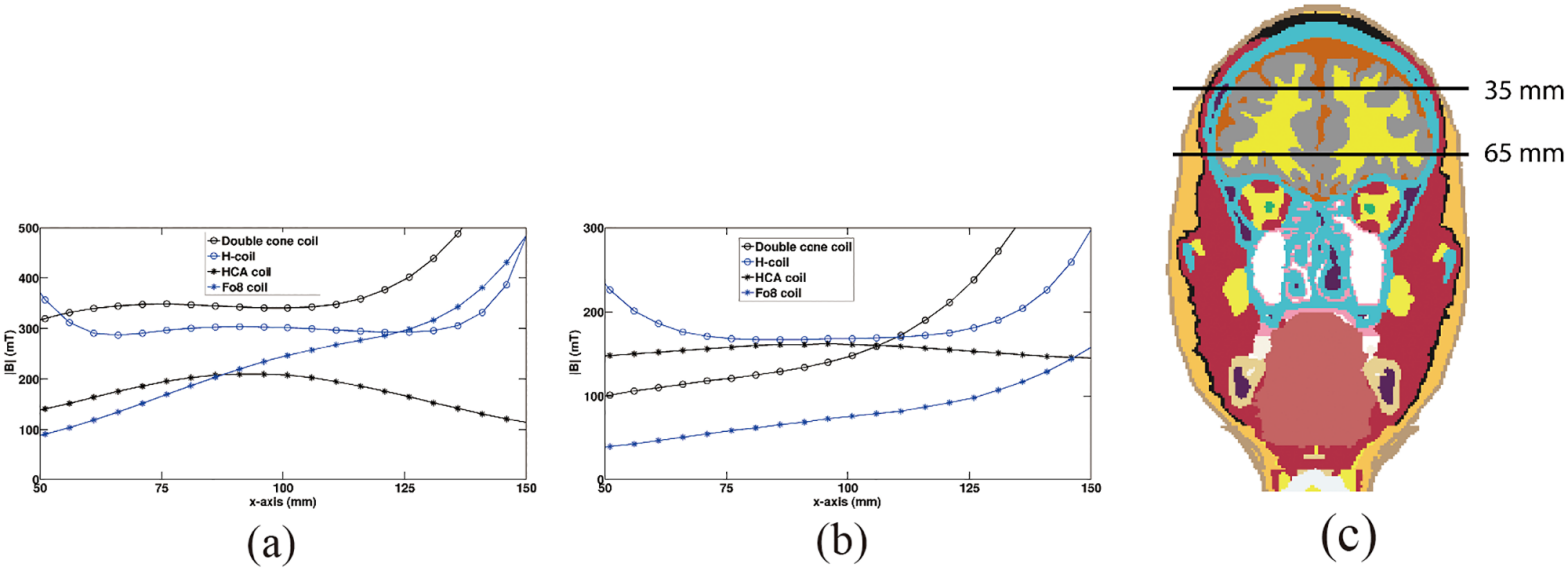
Comparison of B-field along the test lines. (a) test line is located at the depth of 35 mm, (b) test line is located at the depth of 65 mm, and (c) Tissue slice in the coronal plane (y = 80 mm) with two test lines at different depths.

A quantitative comparison of B-field along the test lines at different depths in the same coronal slice of y = 80mm are shown in Fig 4. For the depth of 35 mm (Fig 4(a)), the maximum values of B-filed in brain tissues were obtained by double cone coil. The B-field produced by H-coil is smaller than that of double cone coil, but larger than that of HCA coil. The B-field produced by Fo8 coil is also larger than that of HCA coil in part of the head tissues. For the depth of 65 mm (Fig 4(b)), both H- and HCA coils produced largest B-field in center part of the brain. However, the double cone coil still produced largest B-field in head tissue which were located just under the coil.

The maximum values of B-field in major brain subregions for double cone, H-, HCA and Fo8 coils are presented in Table 2. It was found the high value of magnetic field in brain subregions are always presented by HCA coil.

**Table 2:** Maximum values of magnetic flux density (mT) in deep brain subregions.

	Double cone coil	H-coil	HCA coil	Fo8 coil
Cerebellum	49	75	161	16
Commissure-Anterior	101	130	172	38
Commissure-Posterior	54	98	157	19
Hippocampus	101	98	155	31
Hypophysis	77	104	149	28
Hypothalamus	83	111	156	30
Medalla-Oblongate	22	50	119	8
Midbrain	80	109	162	28
Pinealbody	50	96	157	18
Pons	52	81	140	18
Thalamus	102	134	184	36

**Fig 5:**
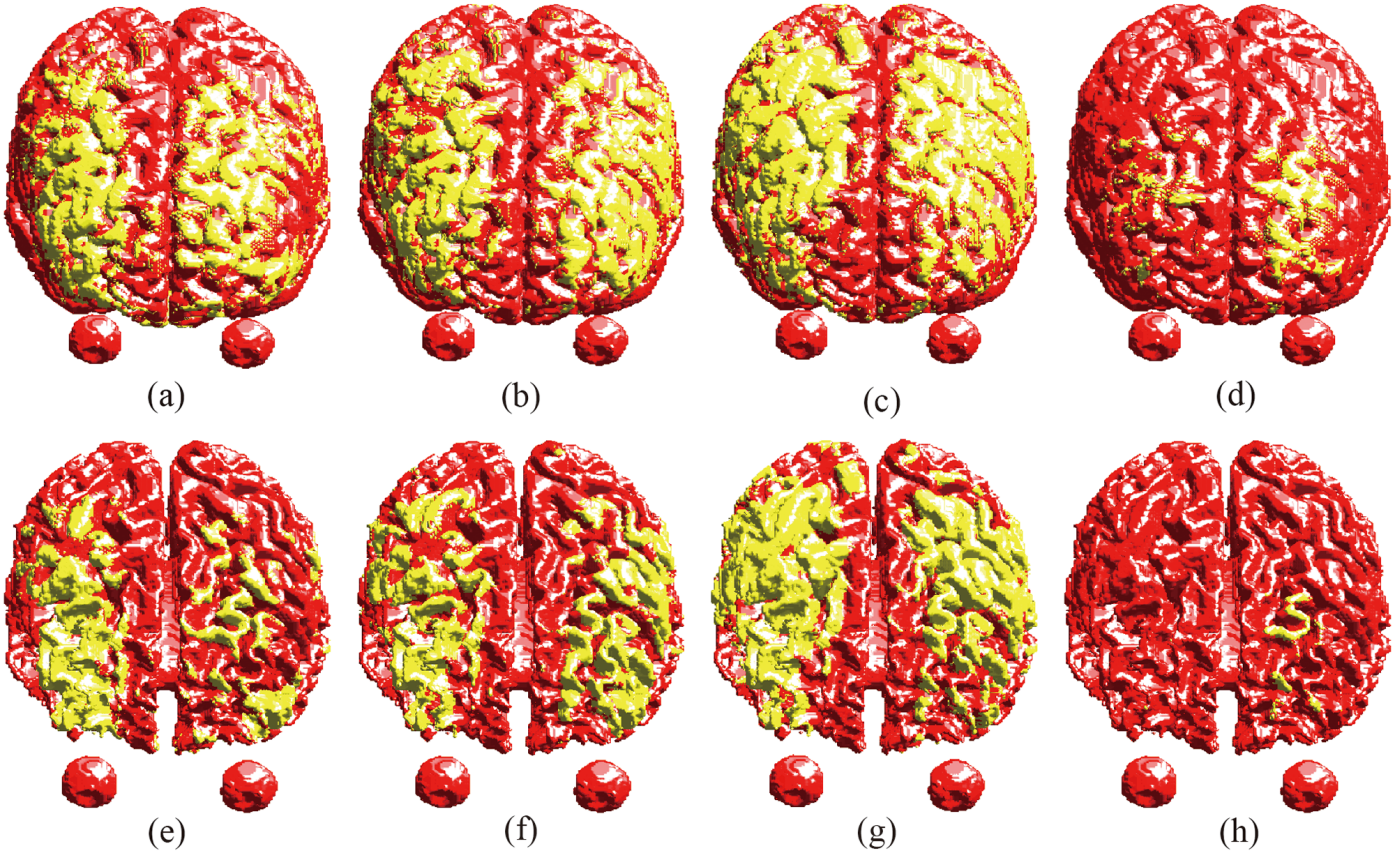
Electric field distributions on the cortical surfaces. Top row: Gray matter, Double cone coil (a), H-coil (b), HCA coil (c) and Fo8 coil (d). Bottom row: White matter, Double cone coil (e), H-coil (f), HCA coil (g) and Fo8 coil (h).

The comparison of the electric field distribution on the surfaces of GM and WM for all four coils are shown in Fig 5. The GM and WM surfaces were represented by red color, while the magnitude of electric field higher than 100 V/m (neuron excitation threshold) was represented by yellow color. It can be found the double cone, H- and HCA coils induce electric fields in wide area on the surfaces of GM and WM. Especially for the HCA coil, it produces wide electric field at the periphery of the GM and WM surfaces. Compared to these dTMS coils, Fo8 coil induces much more focal electric fields.

**Fig 6:**
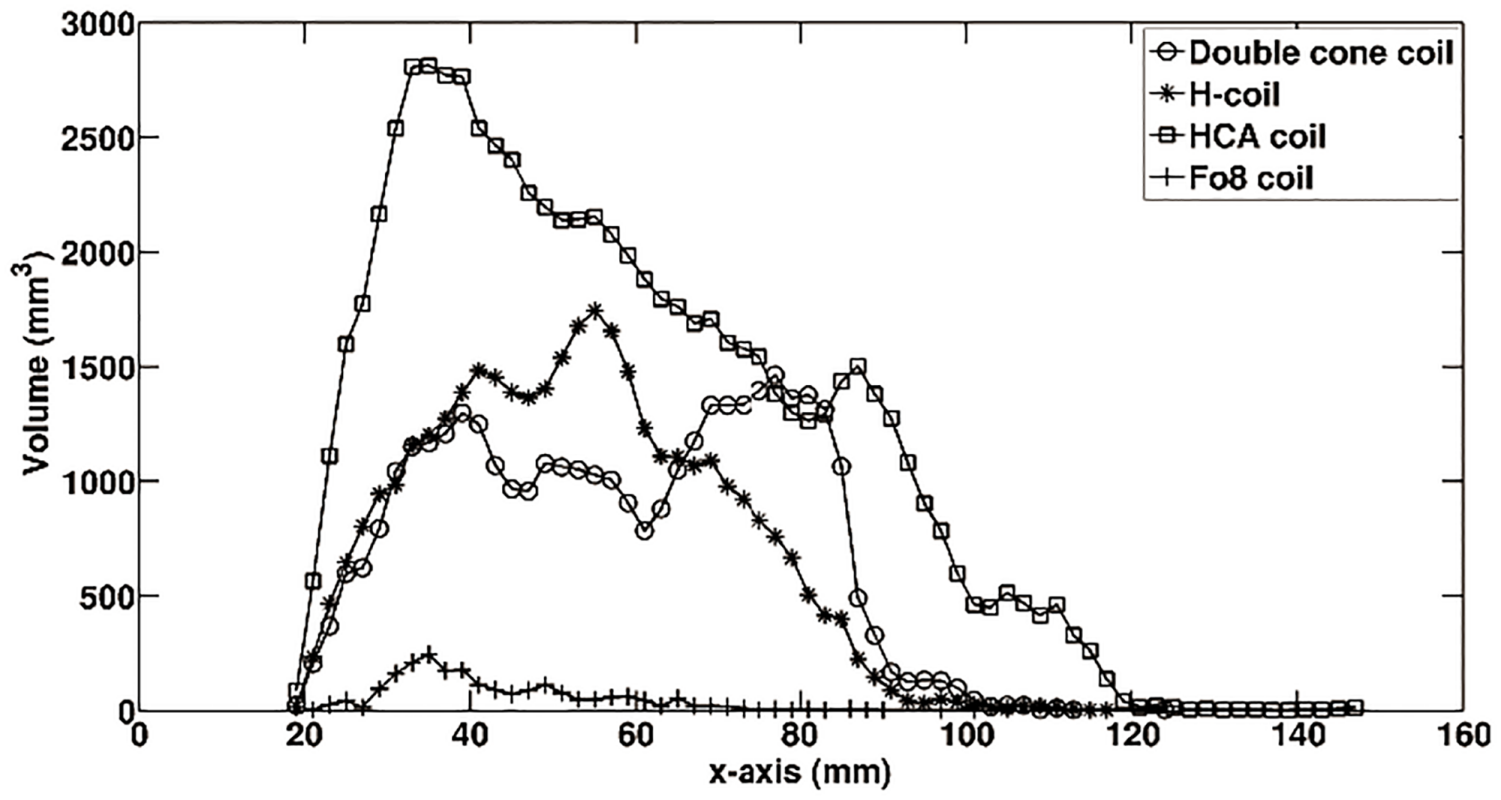
Relationship between the brain tissue volume with E > 100 V/m and the field penetration depths.

Fig 6 shows the dependence of brain volume with electric fields beyond 100 V/m on the distance from the vertex of the head. It can be found the HCA coil has significant depth penetration. It also shows the electric field decays much slower. However, the brain tissue volume potential stimulated by HCA coil is larger compared to both double cone coil and H-coil. It clearly observed the Fo8 coil presents very focal stimulation in superficial cortical regions.

**Fig 7:**
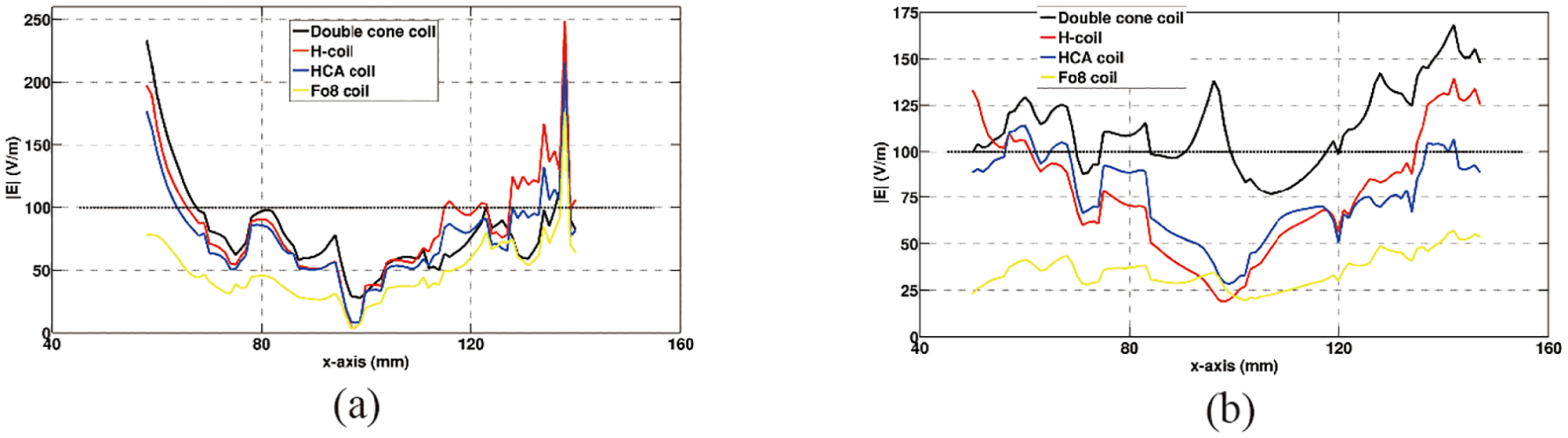
Comparison of electric field in brain tissues at different depth. (a) depth of 35 mm and (b) depth of 65 mm.

Fig 7 shows E-field in brain tissues along the test lines at different depths from the scalp at coronal slice of y = 80 mm in the lateral medial direction for all four coils. It can be found the curves look similar for double cone, H- and HCA coils at the depth of 35 mm (Fig 7(a)). The electric field beyond 100 V/m were presented at the outer region of the brain. However, for the depth of 65 mm (Fig 7(b)), only double cone produces larger electric field beyond 100 V/m in both internal and outer regions of the brain.

Table 3 presents the maximum induced electric field in deep brain subregions by applying the double cone, H-, HCA, and Fo8 coils. It can be found the maximum electric field in brain subregions are produced by either double cone or HCA coil. The H-coil produces smaller electric fields in these deep brain subregions.

**Table 3:** Maximum electric field (V/m) in brain subregions.

	Double cone coil	H-coil	HCA coil	Fo8 coil
Cerebellum	79.1	103.4	319.0	133.3
Commissure-Anterior	85.2	10.3	21.9	22.2
Commissure-Posterior	4.2	6.1	10.1	5.1
Hippocampus	73.8	80.9	120.8	22.5
Hypophysis	129.6	49.6	81.3	43.2
Hypothalamus	134.3	17.6	32.7	35.3
Medalla-Oblongate	16.1	12.9	35.0	2.5
Midbrain	69.9	33.5	56.2	15.3
Pinealbody	15.2	10.0	12.8	4.1
Pons	5.0	18.3	34.6	98.2
Thalamus	86.2	28.7	47.7	21.7

**Fig 8:**
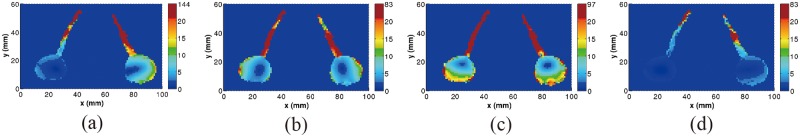
Comparison of the electric field distribution (V/m) in eyeball tissue and optical nerve for double cone coil (a), H-coil (b), HCA coil (c) and Fo8 coil (d).

Since the coils such as H- and HCA coils were placed around the head, close to both eyebows, safety concern about the excitation of visual tissues was therefore arisen. Fig 8 shows the electric field distribution in eyeball and optical nerves by employing all four coils. The slices were position in the cross section plane, 92 mm in depth from the top of the head. It was clearly observed the electric fields in optical nerve is larger than that in eyeball tissues for four coils. For both double cone and HCA coils, since the maximum electric fields in optical nerve is 144 V/m and 97 V/m, respectively, beyond or very close to 100 V/m the neuron stimulation threshold. It suggests the increased risk for optical nerve excitation in using double cone and HCA coil.

## Discussions

TMS is based upon the principle of electromagnetic induction. A stimulation coil produces a transient magnetic field in the brain which induces an electric field in conducting tissue. Most of the previous literatures focused on the induced electric fields in brain tissues. Little information has been published on the distribution of the B-field produced by standard TMS coils except for recently studies [42]. In our present study, we have firstly investigated the spatial variation of B-field produced by dTMS coils. We have observed that all three dTMS coils produce regions of relatively strong B-field in deep brain regions compared with that of Fo8 coil (see Fig 3). This implies that stimulation of deep brain tissues is due to this strong field.

Comparison of induced electric field in spherical head model by dTMS coils have been well addressed by Deng et al [43]. However, since the geometry of human head is obviously significantly different from the spherical head model, the shape of head surface is varied for individualized head model, and the brain tissue heterogeneity and anisotropy were not accounted in the spherical model, the obtained electric field in spherical head model could no represent the real situation. In this study, we presented the comparison of induced electric field in realistic head model by employing double cone coil and H-coil which have been employed in clinical applications for treatment for several neuro-psychiatric disorders, and the HCA coil which is promising for dTMS with flexible structure for adjustable stimulation depth. All these three dTMS coils improve the stimulation depth remarkable at the expense of reduced focality compared to the conventional Fo8 coil (see Fig 5). Especially for the HCA coil, the brain tissue volume with E > 100 V/m is much larger than that of H- and double cone coils (see Fig 6). At depth of 30–40 mm, the brain volume stimulated above threshold are almost same for both double cone and H-coils (see Fig 6). Considering the fact that double cone coil was originally developed to stimulate the leg motor area, which is 3 to 4 cm in depth. It is expected the H-coil also can be employed to stimulate this area. At depth of 40–60 mm, the brain volume stimulated above threshold by H-coil is larger than that of double cone coil. Which implies the stimulation focality of double cone coil is better than that of H-coil at this depth. At depth of 60–80 mm, the brain volume stimulated above threshold by H-coil decreases quickly. While it increases for double cone coil. This result is in agreement with previous study [44], where the authors have found that double cone coil generates a deeper stimulation compared to the H-coil.

Detailed comparison of B-field and E-field in realistic head model between dTMS coils and standard Fo8 coil have been presented in this paper. It was found higher B-field is presented on the coil surface for both dTMS and standard coils. However, the B-field decays slowly away from the coil surface for dTMS coils relative to the Fo8 coil (Fig 3). For this reason, dTMS coils generate stronger electric field that penetrates deeper and stimulates a wider portion of the brain compared to standard Fo8 coil.

There were several limitations in the current study. First, results in this paper are based on the realistic head model with isotropic tissue properties. The tissue anisotropy in particular of the white matter should be incorporated in realistic head model which will show a more precise induced electric field in brain tissues. Second, the distribution of E-field in brain tissues was not optimized as only one set of coil and current parameters were implemented. We plan to study the influence of induced field by varying the coil parameters, their relative positions and different pulse current schemes to improve the stimulation focality in specific deep brain regions that are correlated to specific neural disorders.

## Conclusions

In this paper, typical coil designs such as double cone, H- and HCA coils which were employed for dTMS have been numerically designed. 3D distributions of magnetic flux density, induced electric field in realistic head model using dTMS coils were obtained by the impedance method. Results were compared with that of Fo8 coil. It was found that deeper electric field penetration is obtained by double cone, H- and HCA coils through reducing the rate of decay of the field as a function of distance, which inevitable induce higher and wider spread electrical fields in superficial cortical regions. Both double cone and HCA coils have better ability to stimulate deep brain subregions, but with the risk of optical nerve excitation. The H-coil, on the contrary, has less penetration depth compared with the double cone and HCA coils. In dTMS application, since there exit no coil configuration that is capable of achieving both deep stimulation with improved facolity, the selection of the most suitable coil setting should be based on a balance between the stimulation depth and focality.
